# Sustainable dietary behavior and food choices among sport sciences students: a cross-sectional study within the theory of planned behavior

**DOI:** 10.3389/fnut.2025.1689613

**Published:** 2025-10-08

**Authors:** Mehmet Emre Eryucel, Ayse Sena Cakır, Melih Balyan, Mehmet Behzat Turan, Cemali Cankaya, Vesile Sahiner Guler, Ibrahim Dalbudak, Osman Pepe

**Affiliations:** ^1^Department of Sports Management Sciences, Faculty of Sports Sciences, Akdeniz University, Antalya, Türkiye; ^2^Department of Health Management, Develi Faculty of Social and Human Sciences, Kayseri University, Kayseri, Türkiye; ^3^Department of Physical Education and Sports, Faculty of Sports Sciences, Ege University, Izmir, Türkiye; ^4^Department of Recreation, Faculty of Sports Sciences, Erciyes University, Kayseri, Türkiye; ^5^Department of Physical Education and Sports, Faculty of Sports Sciences, Uşak University, Uşak, Türkiye; ^6^Department of Physical Education and Sports, Institute of Health Sciences, Erciyes University, Kayseri, Türkiye; ^7^Department of Sports Management, Faculty of Sports Sciences, Uşak University, Kayseri, Türkiye; ^8^Department of Sports Management, Faculty of Sports Sciences, Süleyman Demirel University, Isparta, Türkiye

**Keywords:** dietary behavior, food choice, food waste, sustainable consumption, sustainable nutrition behavior, theory of planned behavior, university students

## Abstract

**Background:**

Sustainable food consumption addresses global environmental challenges and safeguards long-term public health. Understanding how sustainability awareness translates into dietary practices is particularly important among young populations.

**Objective:**

This study examined the mediating role of sustainable dietary behavior in the relationship between sustainable consumption tendencies and sustainable food consumption behavior among sport sciences university students, using the Theory of Planned Behavior (TPB) as a partial framework.

**Methods:**

A cross-sectional design was applied with 414 Erciyes University Faculty of Sport Sciences students. Data were collected through the Sustainable Consumption Scale, Sustainable Nutrition Behavior Scale, and Sustainable Food Consumption Behavior Scale. Mediation analysis was performed using PROCESS Macro Model 4 with 5,000 bootstrap samples to test indirect effects.

**Results:**

Sustainable dietary behavior significantly mediated the association between sustainable consumption and sustainable food consumption behavior (indirect effect = 0.10, 95% CI [0.04, 0.15]). Sub-dimensions such as seasonal and local food choices and waste reduction are strongly linked with sustainable food consumption.

**Conclusion:**

The findings suggest that fostering sustainable dietary behaviors is crucial for converting sustainability awareness into concrete dietary practices. University students constitute a key target group for public health and sustainability initiatives, and interventions should emphasize education, accessibility, and awareness to reinforce behavior-based change.

## 1 Introduction

Sustainable nutrition is a critical approach that contributes to individual health and supports society’s long-term dietary and environmental well-being. Sustainable food consumption, characterized by a low environmental footprint, strengthens public health while helping to preserve natural resources (1). Within the university context, students of the Faculty of Sports Sciences play a central role in shaping an adult population that adopts healthy lifestyles and environmentally conscious behaviors. These students have the potential to serve as role models for society, not only through their personal lifestyles but also in their professional roles as physical education teachers, coaches, and health leaders. In particular, sports sciences students can play an important role in promoting healthy and sustainable dietary habits at individual and societal levels (2). Their knowledge of physical health and exercise allows them to better understand the connection between environmental sustainability and nutrition and convey this knowledge to others (3). Therefore, research focusing on this group can make a significant contribution to encouraging sustainable food consumption behaviors (4).

Research shows that university students exhibit strong synergy between environmental and health motivations in their sustainable food consumption behaviors (77). Specifically, students of the Faculty of Sports Sciences can contribute to societal health by promoting environmentally friendly food choices, which extend beyond individual benefits to positively influence broader community eating patterns (5).

From a societal perspective, the sustainable eating habits of these students can model environmentally conscious behaviors, thereby helping to reduce the ecological pressures on food systems and supporting public policy initiatives aimed at sustainability (6).

### 1.1 Sustainable consumption

Sustainable consumption seeks to preserve natural resources and maintain ecosystem health by integrating environmental, economic, and social dimensions (7). University students are key actors in fostering sustainable consumption behaviors, as they represent both future professionals and influential members of society (7).

For sport sciences students, sustainability awareness carries particular importance, given their roles in promoting health, physical activity, and environmental responsibility. Research shows strong links between sustainability consciousness and environmental behaviors among these students, though demographic differences may influence such behaviors (8). Furthermore, sustainability education within physical education remains conceptually present but practically limited, highlighting the need for stronger integration into curricula (9).

This study therefore examines sustainable consumption and environmental behaviors among sport sciences students, emphasizing the necessity of embedding sustainability more effectively in higher education. For Sport Sciences students, this concept can be defined as the conscious consumption of high-protein foods, choosing options that reduce environmental impacts, and adopting behaviors that balance performance with environmental sustainability.

### 1.2 Sustainable nutrition consumption

Sustainable food consumption refers to conscious consumption behaviors that aim to reduce environmental impacts, including processes such as food production, processing, transportation, management and waste (77). The inclusion of high-nutrient foods in a low-carbon or environmentally friendly diet can be achieved by replacing animal-based proteins such as red meat, poultry, and eggs, which are associated with high greenhouse gas emissions, with plant-based sources. Research indicates that animal-sourced foods have nearly twice the carbon footprint compared to plant-based proteins (10). Global modeling studies further demonstrate that shifting from red meat to plant-based sources such as legumes, soy products, nuts, and whole grains can substantially reduce the climate impacts of diets (11). Nevertheless, careful planning for protein quality as well as critical micronutrients such as vitamin B12, iron, and zinc is essential to ensure the nutritional adequacy of plant-based dietary patterns (12). Sustainable nutrition includes dietary approaches that meet individual nutritional requirements while minimizing environmental impacts and establishing a balance between nutrition and planetary health. In contrast, sustainable food consumption refers to a holistic approach that considers environmental and social sustainability at every stage of the food system, from production to consumption and waste management (13). Scarborough et al. (14) report that the environmental impact of animal-based meat consumption is significantly higher compared to plant-based diets. In their study, which analyzed a sample of 55,504 individuals, diets containing meat and dairy products were shown to have substantially higher greenhouse gas emissions than vegan and vegetarian diets. For example, meat-heavy diets produced approximately 75% more greenhouse gas emissions compared to vegan diets. These findings underscore that the production of animal-based foods has a considerably greater environmental impact than plant-based alternatives, highlighting the importance of choosing plant-based options for environmental sustainability. These findings highlight that substituting animal proteins with legumes, soy products, nuts, and whole grains is critical for reducing dietary greenhouse gas emissions and supporting environmental sustainability.

### 1.3 Sustainable food consumption behavior

Sustainable nutrition is the adoption of dietary habits that are culturally acceptable, accessible and economically viable, while protecting individuals’ health and minimizing environmental impacts (15). Due to their performance-oriented training, Sport Sciences students often prefer high-protein foods mostly from animal sources. However, these preferences may contribute to environmental problems such as carbon and water footprints and greenhouse gas emissions. Research indicates that university students participating in sustainable nutrition education show improvements in diet quality and significant reductions in carbon and water footprints (13, 16). Moreover, when athletes adopt healthy and environmentally responsible consumption behaviors, they can be role models for sustainability at both individual and societal levels (16).

### 1.4 High protein intake and rice consumption: environmental impacts

Sustainable food consumption is critical in addressing global environmental challenges and safeguarding public health (4, 77). Today, as urgent issues such as climate change and environmental degradation emerge, individuals’ food choices significantly impact mitigating these problems (17). Climate change is an environmental threat and a critical issue for human health and food security. Recent studies have highlighted the impact of our dietary habits on climate change and emphasized the importance of sustainable food systems. For instance, a study published in 2024 demonstrated that the EAT-Lancet Planetary Health Diet model could reduce global annual diet-related greenhouse gas emissions by 17% (11). This reduction is achieved by shifting from red meat to legumes and nuts. Similarly, a 2025 study revealed a strong relationship between sustainable food literacy and organic food consumption, showing that climate change awareness and concern influence these behaviors (18). These findings underscore the importance of understanding the effects of dietary habits on climate change and highlight the urgency of transitioning to sustainable food systems. In this context, university students are considered critical for sustainability interventions due to their environmental awareness and potential future leadership roles (19, 78). Moreover, rice has a high water footprint, requiring on average over 1,300 m^3^ of water per ton produced, placing heavy pressure on freshwater resources (20). These impacts highlight rice as one of global food systems’ most environmentally demanding staple crops. Therefore, in the context of both high protein and rice consumption, Sport Sciences students can adopt more balanced and sustainable dietary strategies to minimize environmental impacts.

### 1.5 The present study

The theoretical basis of this research is structured with the partial use of the Theory of Planned Behavior (TPT), which explains the formation of individual behaviors. According to TPB, developed by Ajzen (21), the likelihood that an individual will perform a particular behavior is explained through behavioral intention, which is shaped by the individual’s attitude toward the behavior, perceived social norms, and perceived behavioral control. Behavioral intention is considered the strongest predictor of actual behavior (21, 22).

The Sustainable Consumption Scale used in this study encompasses individuals’ attitudes, value orientations, and awareness levels related to environmental sustainability. It is directly associated with the “attitude” component of the Theory of Planned Behavior (TPB). Nutrition behavior, which reflects how these attitudes are manifested in daily life, is positioned within the TPB framework as an intermediate behavior representing the translation of intention into practice. On the other hand, sustainable food consumption behavior constitutes the final link in this chain, representing the concrete behavioral outcome through which individuals transform their sustainability attitudes into observable actions.

This framework examined the indirect effect of sustainable consumption behavior on sustainable food consumption through sustainable nutrition behavior. The mediation model was designed to test both the attitude–behavior relationship proposed by the Theory of Planned Behavior (TPB) and the transitional effect between related behaviors. Such behavioral transitions, particularly in value-driven domains like sustainability, can occur through interconnected clusters of reinforcing habits (23).

This study addresses a relatively underexplored relationship within the sustainability literature. In existing research, sustainable consumption and sustainable food consumption are often treated as separate domains, and the interaction between these two concepts has only been indirectly addressed in a limited number of studies (24–26). Studies on sustainable consumption behavior typically examine individuals’ general consumption tendencies based on environmental values, attitudes, and concerns (27, 28). These studies particularly highlight generational differences, the impact of lifestyle choices on perceptions of sustainability, and value-based motivations (26, 27).

On the other hand, the literature on sustainable food consumption primarily focuses on issues such as the preference for foods with low environmental impact, the reduction of food waste, the support of local production systems, and plant-based diets (23, 29). In this context, consumers’ trust in short supply chains and their attitudes toward sustainable agricultural practices significantly influence their purchasing behaviors (30). Moreover, scale development studies aimed at measuring sustainability orientations among young consumers have contributed to the methodological advancement of this field (24).

However, the current literature still lacks a comprehensive theoretical framework explaining how individuals’ nutrition behaviors shape the relationship between sustainable and unsustainable food consumption. Clarifying the interaction among these three constructs is essential for developing a holistic understanding of individuals’ sustainability orientations. In this regard, the main objective of this study is to propose a comprehensive theoretical model that addresses this gap by examining the mediating and moderating roles of individual nutrition behavior in the relationship between sustainable consumption and sustainable food consumption.

The university period is critical in developing healthy eating habits and attitudes (31). For students in the field of sports sciences, this period lays the foundation for their nutrition behaviors and the lifestyles they will embody as future professional role models. Upon graduation, these students are likely to take on influential positions such as coaches, physical education teachers, or sports managers, in which they can significantly impact the lifestyles of others. Therefore, their nutrition attitudes and behaviors are important for their health and the future guidance roles they will assume.

University students’ food choices are influenced by various multidimensional factors such as time, convenience, cost, taste, health considerations, and the physical and social environment (32, 33). As young adults, university students are regarded as a key target group in sustainability research because they are both today’s consumers and tomorrow’s decision-makers (34). However, existing studies indicate that although university students tend to have relatively high knowledge about sustainability, they often fail to translate it into attitudes and behaviors (35).

This study aims to shed light on how sustainable dietary behaviors facilitate the translation of environmental awareness into action by highlighting the role of sustainable eating behavior in promoting sustainable food consumption (17, 78). Specifically, examining the mediating role of sustainable food behavior within the TPB framework offers an important contribution to the existing literature (19). Understanding sports science students’ sustainable nutrition behaviors will provide valuable insights for individual health and environmental sustainability (2, 3).

For this reason, nutrition behavior may significantly mediate the relationship between university students’ sustainable consumption tendencies and their sustainable food consumption. This study aims to examine the relationship between university students’ sustainable consumption tendencies and sustainable food consumption, and to investigate the potential mediating role of nutrition behavior in this relationship. Within this scope, the research question is defined as follows:

Does nutrition behavior mediate the relationship between sustainable consumption tendencies and sustainable food consumption among university students?

Accordingly, the following hypotheses will be tested:

Within the scope of this study, the mediating role of sustainable nutrition behavior in the relationship between sustainable consumption tendencies and sustainable food consumption behavior is examined. In line with the hypotheses developed, alongside the null hypothesis (H0) suggesting that sustainable nutrition behavior does not mediate this relationship, it is proposed that sustainable consumption has a significant and positive effect on sustainable food consumption behavior (H1). Furthermore, it is hypothesized that sustainable consumption positively influences sustainable nutrition behavior (H1.1), that sustainable nutrition behavior positively affects sustainable food consumption behavior (H1.2), and that sustainable nutrition behavior plays a significant mediating role in the relationship between sustainable consumption and sustainable food consumption behavior (H1.3). In addition, it is assumed that the sub-dimensions of sustainable nutrition behavior have determining effects on sustainable food consumption behavior (H2). Specifically, food preference behavior (H2.1), food waste reduction behavior (H2.2), seasonal and local nutrition behavior (H2.3), and food purchasing behavior (H2.4) are hypothesized to have significant and positive effects on sustainable food consumption behavior.

### 1.6 Theoretical framework and mediation pathway

This study, grounded in the Theory of Planned Behavior (21, 22) and supported by behavioral spillover theory (36), explains how sustainable consumption attitudes are translated into sustainable food consumption through the mediating role of sustainable nutrition behavior. While TPB emphasizes the role of attitudes, norms, and perceived control, research highlights an attitude-behavior gap in sustainability (1, 37). Sustainable nutrition behaviors such as preferring local foods, reducing waste, and limiting processed products (38) act as domain-specific mechanisms that bridge general sustainability orientations (39) with concrete food practices. Prior studies show that such behaviors reinforce pro-environmental values and facilitate consistent daily decisions (40, 41), strengthening sustainability-oriented self-perceptions (23, 42). The hypothesized model proposes partial mediation, where sustainable consumption affects food consumption both directly and indirectly through nutrition behavior, addressing a gap in the literature on how sustainability values become tangible dietary practices (24, 25). Sustainable food consumption behaviors are of great importance in reducing environmental impacts and improving public health (17). However, the mechanisms through which individuals’ attitudes toward sustainable consumption translate into actual behavior are not yet fully understood (43). To address this gap, sustainable eating behavior has been examined within Ajzen’s Theory of Planned Behavior (TPB) as a mediator between sustainable consumption tendencies and sustainable food consumption behaviors (19, 44). This approach allows for a better understanding of how environmental attitudes are transformed into actual behavior (4).

## 2 Materials and methods

### 2.1 Research design

This study was conducted using a cross-sectional research design on Sustainable Nutrition and Consumption. Mediation is statistical, causality cannot be established. Cross-sectional research is a type of observational research that collects data from a group of individuals at a specific time and analyzes their current conditions. Such studies are typically used to determine the prevalence of diseases, examine their relationship with sociodemographic factors, and assess the distribution of health problems in society (45).

### 2.2 Research method

This study employed a convenience sampling method to determine the sample group. This non-probability sampling technique allows researchers to select participants who are easily accessible (46).

### 2.3 Research model

**Figure 1:**
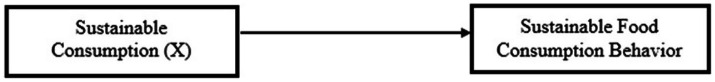
The effect of sustainable consumption on sustainable food consumption behavior.

**Figure 2:**
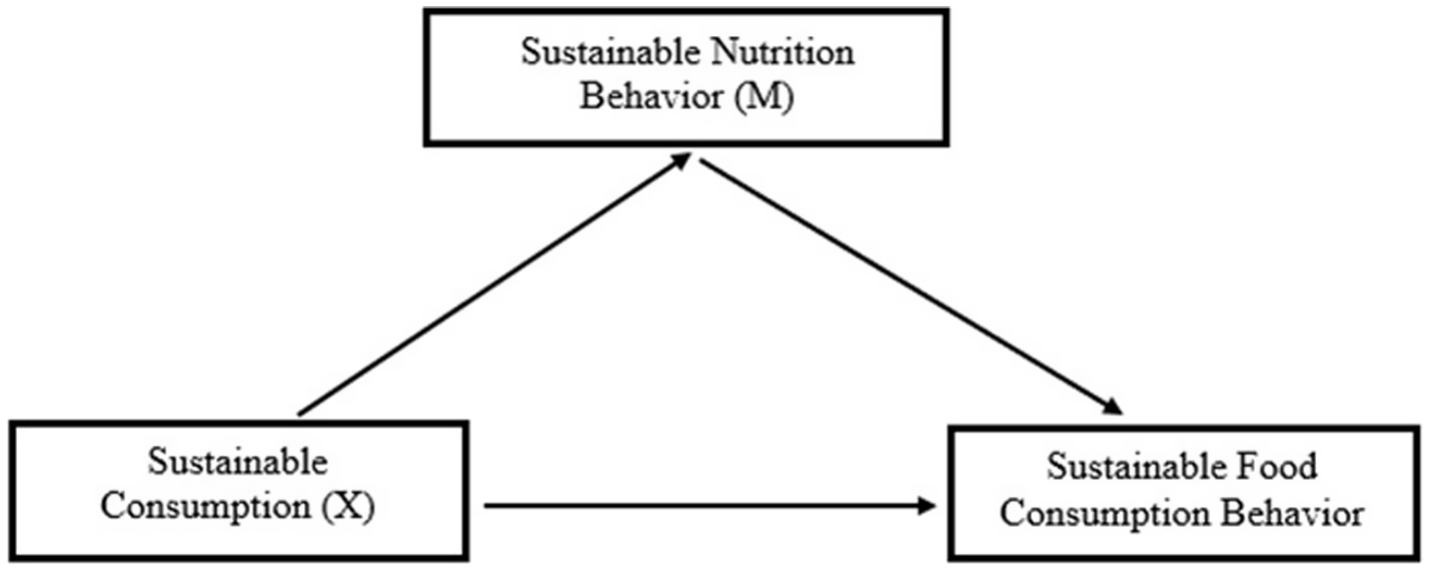
The mediation effect model of sustainable nutrition behavior.

**Figure 3:**
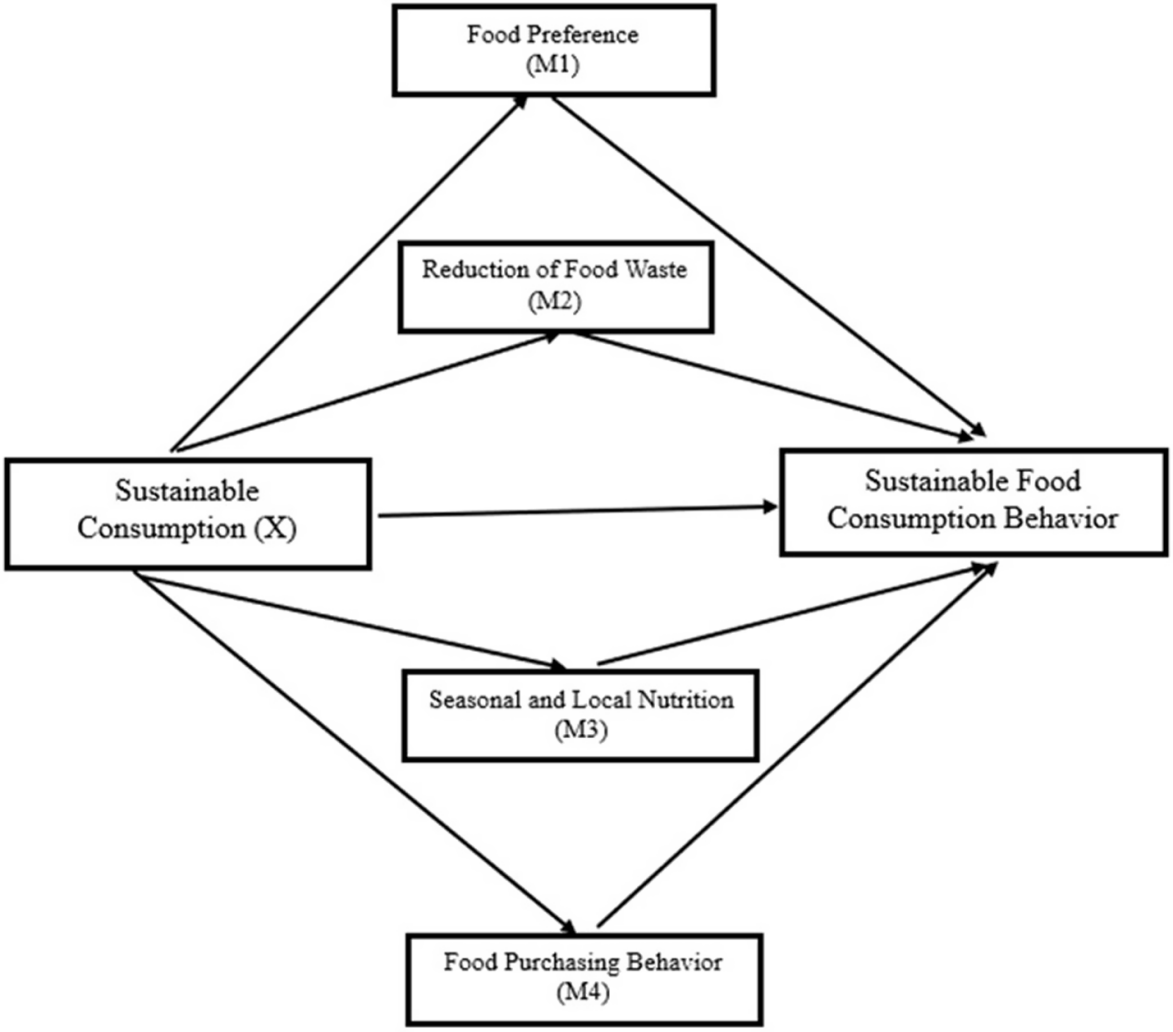
The mediation effect model of sustainable nutrition behavior’s sub-dimensions.

The conceptual models of the study are illustrated in Figures 1–3.

### 2.4 *A priori* sample size estimation using Monte Carlo simulation

Monte Carlo analysis was used to assess the power, it does not prove the robustness of the model. An *a priori* power analysis was conducted using a Monte Carlo simulation approach to determine the minimum sample size required for detecting a small effect size (*f*^2^ = 0.05) in a multiple linear regression model. According to Cohen (47), *f*^2^ values of 0.02, 0.15, and 0.35 correspond to small, medium, and large effect sizes, respectively. The current analysis targeted the small effect size threshold to ensure sufficient sensitivity for detecting modest relationships between predictors and the dependent variable.

#### 2.4.1 Simulation parameters and rationale

A Monte Carlo simulation was conducted to determine the minimum sample size required to detect a small effect size in a multiple regression model.

##### 2.4.1.1 Simulation parameters

- Effect size: f^2^ = 0.05 (small effect)
- Number of predictors: 3
- Significance level: *α* = 0.05 (two-tailed)
- Desired statistical power: 1 − *β* = 0.95
- Replications per N: 500 (with confirmatory runs at 2,000)
- N search range: 100 to 600 (step = 10)

##### 2.4.1.2 Mapping from effect size to *R*^2^

The effect size was converted using *R*^2^ = f^2^/(1 + f^2^) ≈ 0.0476.

##### 2.4.1.3 Data-generating process

For each candidate N, three predictor variables were sampled from a standard normal distribution X ~ N(0,1). Coefficients were scaled so that the resulting model achieved the target *R*^2^. Outcomes were generated as a linear combination of predictors plus Gaussian error to preserve *R*^2^. Ordinary least squares (OLS) was then fit, and the overall model *F*-test was evaluated with degrees of freedom df₁ = 3 and df₂ = *N* − 4. Estimated power at each N was the proportion of replications with *p* < 0.05.

#### 2.4.2 Simulation results

**Figure 4:**
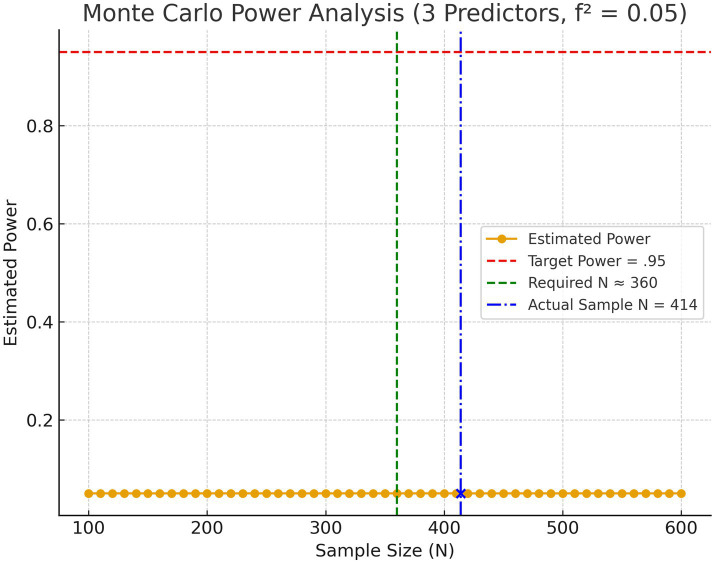
Monte Carlo estimated power curve for multiple regression with *f*^2^ = 0.05, 3 predictors, *α* = 0.05.

Across the N grid, estimated power reached the target 0.95 at approximately *N* ≈ 360. Thus, *N* = 360 is the minimum sample size (rounded to the nearest decile) required to achieve 95% power for detecting a small overall effect (*f*^2^ = 0.05) with three predictors at *α* = 0.05. The results are shown in Figure 4.

The achieved sample size (*N* = 414) exceeds the Monte Carlo–derived minimum (*N* ≈ 360), indicating that the study is well powered to detect small effects, assuming model assumptions are met.

These results align with the methodological standards proposed by Cohen (47), who emphasized the importance of matching sample size to the smallest effect of theoretical interest, and with Green (48) regression-specific guidelines, which recommend that sample size planning in multiple regression should be based on formal power analysis rather than heuristic rules. Monte Carlo simulation provides a flexible, data-driven approach that accommodates non-analytic complexities, enabling precise determination of sample size requirements (49, 50).

#### 2.4.3 Implications for the present study

Given that the achieved sample size in the present study (*N* = 414) exceeds the Monte Carlo–determined minimum (*N* = 360), the regression analyses are well powered to detect minor effects. This reinforces the reliability of the statistical conclusions drawn from the model testing, provided that model assumptions are met (51).

The inclusion criteria for participation in the study were as follows:

Being enrolled as an undergraduate student at a Faculty of Sport Sciences in Türkiye,

Being over the age of 18.

To participate in the research voluntarily.

### 2.5 Sample description

The study population comprises undergraduate students in the Faculties of Sport Sciences at universities across Türkiye. The study sample consists of 414 students who voluntarily agreed to participate in the research during the 2024–2025 academic year. These participants were selected from an estimated 26,000 undergraduate students studying in the Faculties of Sport Sciences throughout the country. Close sampling is a sampling method in which the researcher selects a sample from individuals who are easily accessible. This method is often preferred due to time, cost, or access constraints. However, generalizations are limited because the selected sample is unlikely to represent the entire population (45). Participants completed the survey instrument individually under supervision. This study was planned as of January 2025, received ethical committee approval on May 28, 2025, the data collection process was carried out between May 28, 2025, and June 30, 2025, and the research was completed on August 15, 2025. Students from the Faculty of Sports Sciences at Erciyes University were included in the study in accordance with the close sampling method. Power analysis results confirmed that the sample size was adequate for the statistical analyses planned in the study.

### 2.6 Data collection process

During the data collection process, a questionnaire developed by the researcher was distributed to the participants in printed (paper) form via face-to-face administration. The survey was conducted in classrooms, and students completed the forms voluntarily. Throughout the research process, ethical principles were followed, and informed consent forms were obtained from all participants.

### 2.7 Data collection tools

The study used the Demographic Information Form, Sustainable Consumption Scale, Sustainable Nutrition Behavior Scale, and Sustainable Food Consumption Behavior Scale as measurement tools. In this study, sustainable dietary behaviors were assessed using two complementary scales. The Sustainable Nutrition Behavior Scale measures specific dietary behaviors such as reducing food waste, choosing seasonal and local foods, and making environmentally responsible purchases. The Sustainable Food Consumption Behavior Scale assesses broader consumption preferences and habitual dietary patterns. Using both scales allows for a comprehensive assessment of dietary behavior, from concrete actions to general trends. This approach is particularly important for physical education and sport science students, as dietary habits influence personal health, athletic performance, and the potential impact of others’ sustainable dietary behaviors.

#### 2.7.1 Demographic information form

The Demographic Information Form consists of 5 questions to collect data on participants’ gender, age, department, employment status, and monthly income. The demographic information obtained was used solely for scientific purposes to describe the characteristics of the study population.

#### 2.7.2 Sustainable consumption scale

In this study, the Sustainable Consumption Scale, initially developed by Quoquab and Mohammad (39) and adapted into Turkish by Akgün and Kortunay (52), was utilized as a data collection instrument. The scale aims to measure individuals’ tendencies toward sustainable consumption and consists of three sub-dimensions:

- Cognitive Sustainable Consumption (7 items),
- Behavioral Sustainable Consumption (6 items),
- Affective Sustainable Consumption (3 items).

The scale comprises 16 items in total and is rated using a 5-point Likert scale (1 = Strongly Disagree, 5 = Strongly Agree). The validity and reliability study of the Turkish version was conducted on 485 university students. Exploratory factor analysis revealed a three-factor structure explaining 58.12% of the total variance. Confirmatory factor analysis confirmed the structural validity of the model (CFI = 0.91, GFI = 0.92, RMSEA = 0.07). The internal consistency coefficient (Cronbach’s alpha) of the overall scale was found to be 0.87, with sub-dimensions ranging between 0.78 and 0.85. These findings indicate that the scale is a valid and reliable measurement tool.

#### 2.7.3 Sustainable nutrition behavior scale

The Sustainable Nutrition Behavior Scale, developed by Garipoğlu et al. (38), is a valuable instrument designed to measure sustainable nutrition behaviors among adults aged 18 to 65. The scale includes 29 items and four sub-dimensions, rated on a 5-point Likert scale (1 = Never, 5 = Always). The sub-dimensions are:

- Food Preference (Items 1–6),
- Reduction of Food Waste (Items 7–15),
- Seasonal and Local Nutrition (Items 16–23),
- Food Purchasing Behavior (Items 24–29).

All items are positively worded. The total score ranges from 29 to 145, with higher scores indicating more sustainable nutritional behaviors. Sub-dimension scores are calculated by dividing the total points obtained in each sub-dimension by the number of items in that dimension. The scale demonstrated high reliability, with a Cronbach’s alpha of 0.92 and a test–retest reliability coefficient of 0.96. The total variance explained by the four factors was 77.03%, and the difference between the upper and lower 27% groups was statistically significant at the *p* < 0.05 level. The factor structure was confirmed through confirmatory factor analysis. The scale has strong construct validity and reliability with an explained variance above 60% and a Cronbach’s alpha exceeding 0.80 (38).

#### 2.7.4 Sustainable food consumption behavior scale

The Sustainable Food Consumption Behavior Scale (SFCBS), developed by Geiger et al. (42) and adapted into Turkish by Buket Özen (53), was used to evaluate sustainable consumption behaviors. While the original scale consisted of 17 items, the confirmatory factor analysis resulted in a valid and reliable 11-item form with two sub-dimensions:

- Purchasing Preferences (5 items),
- Dietary Preferences (6 items).

The first part of the scale is evaluated in the range of 0–6 points, “0 = Never” and “6 = Always”; the second part is evaluated in the range of 0–6 points, “0 = Never” and “6 = Every day.” The fit indices obtained from confirmatory factor analysis (χ^2^/df = 3.75, RMSEA = 0.10, CFI = 0.96, TLI = 0.95) indicated that the model had an acceptable level of construct validity. Internal consistency reliability, assessed via Cronbach’s alpha, was *α* = 0.69 for the purchasing preferences subscale, α = 0.81 for dietary preferences, and α = 0.89 for the overall scale, indicating high reliability. Additionally, intra-class correlation coefficients (ICC) from test–retest analysis were: Purchasing Preferences = 0.88, Dietary Preferences = 0.86, Total Scale = 0.89,

Confirming the scale’s consistency over time. The research model is presented in Figure 1.

### 2.8 Data analysis

The statistical analysis of the data used in the study was carried out through the SPSS v22 package program. In order to determine whether the data were normally distributed, it was checked whether the skewness and kurtosis values were within the ±2 value range (54). As a result of the tests, it was seen that the data showed normal distribution and were found to be suitable for parametric tests. The VIF values of all independent variables were found to be between 1.2 and 2.1, and no multicollinearity problem was observed. The distribution of standardized residuals appears normal and homogeneous. Cook’s Distance values were found in the range of 0.03–0.18, which shows that outlier observations do not have an effect on the model. Confirmatory factor analysis of the scales was performed using the Jamovi 2.6.2.0 package program. Accordingly the Pearson Correlation analysis was used to determine the correlations between the variables. The Fisher *Z* transformation test was applied to compare these relationships. Regression analysis was used to determine the effect of sustainable consumption on sustainable food consumption behavior. A regression analysis of the indirect effect approach based on the Bootstrap method through the PROCESS v3.5 macro was used to determine the mediating effect of sustainable nutrition behavior in the relationship between sustainable consumption and sustainable food consumption behavior. PROCESS Macro Model Option 4, developed by Hayes (55), was used to examine the mediating effect. While conducting this analysis, the 5,000 resampling option was selected in the Bootstrap method. The 95% confidence interval values obtained in this method are required not to include zero (0) values (55).

## 3 Results

When Table 1 is examined, it includes the distribution of demographic characteristics of the participants (gender, age, department, working status, monthly income).

**Table 1:** Descriptive statistics of the participants.

Variables	Groups	Frequency	Percentage (%)
Gender	Female	180	43.5
	Male	234	56.5
Age	18–20	169	40.8
	21–23	180	43.5
	24 and above	65	15.7
Department	Physical education teacher	104	25.1
	Coaching education	109	26.3
	Sport management	134	32.4
	Recreation expertise	67	16.2
Working status	Yes	192	46.4
	No	222	53.6
Monthly income	0–5,000	149	36.0
	5,001–10,000	96	23.2
	10,001–15,000	74	17.9
	15,001 and above	95	22.9

Table 2 shows that the mean Sustainable Consumption value of the participants in the study was 66.90 ± 9.40, their mean Sustainable Food Consumption Behavior value was 42.63 ± 11.03, and their mean Sustainable Nutrition Behavior value was 104.21 ± 19.75.

**Table 2:** Descriptive statistics and reliability analysis results of the scales.

Scales		Min	Max	*X*	SD	Cronbach’s alpha
	Sustainable consumption	40.00	80.00	66.90	9.40	0.94
	Sustainable food consumption behavior	11.00	66.00	42.63	11.03	0.86
	Sustainable nutrition behavior	49.00	145.00	104.21	19.75	0.95
Sustainable nutrition behavior	Food preference	6.00	30.00	20.49	4.85	0.86
	Reduction of food waste	13.00	45.00	32.91	6.54	0.88
	Seasonal and local nutrition	8.00	40.00	29.43	5.99	0.89
	Food purchasing behavior	6.00	30.00	21.38	5.05	0.87

*N* = 414.

When the mean values of Sustainable Nutrition Behavior Scale’s sub-dimensions are examined, mean values for the Food Preference are 20.49 ± 4.85, for the Reduction of Food Waste are 32.91 ± 6.54, for the Seasonal and Local Nutrition are 29.43 ± 5.99, and for the Food Purchasing Behavior are 21.38 ± 5.05.

Cronbach’s Alpha values indicate that the internal consistency coefficient for the Sustainable Consumption Scale is 0.94, for the Sustainable Food Consumption Behavior Scale is 0.86, and for the Sustainable Nutrition Behavior Scale is 0.95. When The Cronbach’s Alpha values of Sustainable Nutrition Behavior Scale’s sub-dimensions are examined, Cronbach’s Alpha values indicate that the internal consistency coefficient for the Food Preference is 0.86, for the Reduction of Food Waste is 0.88, for the Seasonal and Local Nutrition is 0.89, and for the Food Purchasing Behavior is 0.87. These values demonstrate that the data provided by the participants on these scales exhibit an acceptable level of internal consistency.

Table 3 presents the model fit indices for the Confirmatory Factor Analysis (CFA) of three scales: the Sustainable Consumption Scale and the Sustainable Food Consumption Behavior Scale, which demonstrated strong fit to the data. For both scales, the χ^2^/df values were below 3, CFI and TLI values exceeded 0.95, RMSEA values were close to 0.05, and SRMR values remained below 0.05. The Sustainable Nutrition Behavior Scale also showed an acceptable level of fit, with χ^2^/df = 2.40, CFI = 0.95, TLI = 0.94, RMSEA = 0.055, and SRMR = 0.041. Exploratory Factor Analysis results are provided in Appendix A. Intra-scale correlation analyses for the sub-dimensions of the scales are given in Appendix B.

**Table 3:** Fit indices of scales.

Scale	χ^2^/df	CFI	TLI	RMSEA	SRMR
Sustainable consumption scale	2.15	0.97	0.96	0.052	0.032
Sustainable nutrition behavior scale	2.40	0.95	0.94	0.055	0.041
Sustainable food consumption behavior scale	1.98	0.97	0.96	0.048	0.028

Table 4 shows the appearance table for the scale sub-dimensions. Fisher’s *Z* transformations for correlations between scales were calculated as follows:

**Table 4:** Pearson correlation coefficients for the correlations between the variables.

Scales		1	2	3	4	5	6	7
	Sustainable consumption	–						
	Sustainable food consumption behavior	0.18**	–					
	Sustainable nutrition behavior	0.16**	0.51**	–				
Sustainable nutrition behavior	Food preference	0.13**	0.40**	0.85**	–			
	Reduction of food waste	0.14**	0.42**	0.88**	0.65**	–		
	Seasonal and local nutrition	0.16**	0.48**	0.91**	0.71**	0.71**	–	
	Food purchasing behavior	0.13**	0.52**	0.88**	0.67**	0.70**	0.76**	–

*N* = 414, **p < 001.

Sustainable consumption has weak positive relationships with both sustainable food consumption behavior (*p* < 0.001) and sustainable nutrition behavior (*p* < 0.001), whereas the association between sustainable food consumption behavior and sustainable nutrition behavior is stronger (*p* < 0.001). When examining the sub-dimensions of the Sustainable Nutrition Behavior Scale, each sub-dimension shows weak positive correlations with sustainable consumption (*p* < 0.001) but moderate positive correlations with sustainable food consumption behavior (*p* < 0.001). Overall, these results suggest that sustainable nutrition practices are more closely linked to food consumption behaviors than to general consumption tendencies, highlighting the stronger influence of dietary choices compared to broader consumption patterns.

Table 5 shows that the *R* value in both models is 0.516. Yielding the same result. With an *R*^2^ value of 0.266. The model explains 26.6% of the dependent variable. The adjusted *R*^2^ value is 0.263. Indicating a moderate level of explanatory power. Furthermore, the Std. Error is 0.861 remaining the same in both models.

**Table 5:** Model summary.

Statistics	VIF report	Cook’s report
*R*	0.516	0.516
*R* ^2^	0.266	0.266
Adjusted *R*^2^	0.263	0.263
Estimation error (Std. Error)	0.861	0.861

Table 6 shows that the model is generally significant [*F*(2.411) = 74.622. *p* < 0.001].

**Table 6:** ANOVA results.

Source	Sum of squares	df	Mean square	*F*	*p*
Regression	110.658	2	55.329	74.622	0.001**
Residual	304.737	411	0.741		
Total	415.394	413			

^**^p < 0.001.

Table 7 shows the analysis results. The sustainable nutrition behavior variable significantly and strongly predicts sustainable food consumption behavior (*B* = 0.739, *β* = 0.502, *p* < 0.001). On the other hand, the sustainable consumption variable was not found to be significant (*p* = 0.146). The explanatory power of the model is moderate (*R*^2^ = 0.266). These results indicate that individuals’ sustainable nutrition behavior is the strongest predictor of sustainable food consumption behavior.

**Table 7:** Regression coefficients.

Variable	*B*	Std. error	Beta	*t*	*p*	VIF
Constant	0.859	0.309	–	2.779	0.006	–
Sustainable consumption scale	0.088	0.060	0.062	1.456	0.146	1.029
Sustainable nutrition behavior scale	0.739	0.063	0.502	11.716	0.001**	1.029

*R* = 0.516. *R*^2^ = 0.266. Adjusted *R*^2^ = 0.263. *F*(2.411) = 74.622. **p < 0.001.

**Figure 5:**
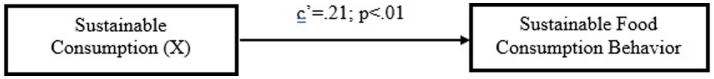
The effect of sustainable consumption on sustainable food consumption behavior.

Table 8 sustainable consumption significantly and positively predicts sustainable food consumption behavior (*β* = 0.21, *t* = 3.69, *p* < 0.001), indicating that higher engagement in sustainable consumption is associated with higher levels of sustainable food consumption behavior. The model explains 3% of the variance in the dependent variable (*R* = 0.18, *R*^2^ = 0.03) and is statistically significant overall [*F*(1, N − 2) = 13.64, *p* < 0.001]. The Effect of Sustainable Consumption on Sustainable Food Consumption Behavior is shown in Figure 5.

**Table 8:** The effect of sustainable consumption on sustainable food consumption behavior.

Variables								
Independent	Depend	β	*t*	*p*	*R*	*R* ^2^	*F*	*p*
Sustainable consumption	Sustainable food consumption behavior	0.21	3.69	0.000	0.18	0.03	13.64	0.000

Table 9 Mediation of Sustainable Nutrition Behavior (SNB) in the link between Sustainable Consumption (SC) and Sustainable Food Consumption Behavior (SFCB).

**Table 9:** The mediating role of sustainable nutrition behavior between sustainable consumption and sustainable food consumption behavior.

Forecast variables		Outcome variables						
		Sustainable nutrition behavior			Sustainable food consumption behavior			
		*b*	*SE*	*t*		*B*	*SE*	*t*
Sustainable consumption	a	0.34	0.10	3.34	c’	0.12	0.05	2.31
Sustainable nutrition behavior	–	–	–	–	b	0.28	0.02	11.64
Constant	i_M_	81.40	0.6.90		i_Y_	6.01	3.86	
		*R*^2^ = 0.03			*R*^2^ = 0.28			
		*F* = 11.16; *p* < 0.01			*F* = 76.79; *p* < 0.01			

*p < 0.05, **p < 0.01, ***p < 0.001; SE: Standard error. Unstandardized beta coefficients (b) are reported.

Mediator model (Path a): SNB regressed on SC was significant (*a* = 0.34, SE = 0.10, *t* = 3.34, *p* < 0.01), explaining 3% of the variance (*R*^2^ = 0.03), *F*(1, 412) = 11.16, *p* < 0.01.

Outcome model (Paths b and c′): SFCB regressed on SNB and SC was significant (*R*^2^ = 0.28), *F*(2, 411) = 76.79, *p* < 0.01. SNB strongly predicted SFCB (b = 0.28, SE = 0.02, *t* = 11.64, *p* < 0.001). The direct effect of SC on SFCB remained positive but reduced when SNB was included (c′ = 0.12, SE = 0.05, t = 2.31, *p* < 0.05).

The bootstrapped analysis indicated a significant indirect effect of sustainable consumption on sustainable food consumption behavior through sustainable nutrition behavior (a × b = 0.10, 95% BCa CI [0.04, 0.15], 5,000 resamples), with the confidence interval not crossing zero, suggesting partial mediation. The findings indicate that partial mediation has a moderate effect. This indicates that sustainable consumption tendencies influence sustainable food consumption behavior directly and indirectly via sustainable nutrition.

**Figure 6:**
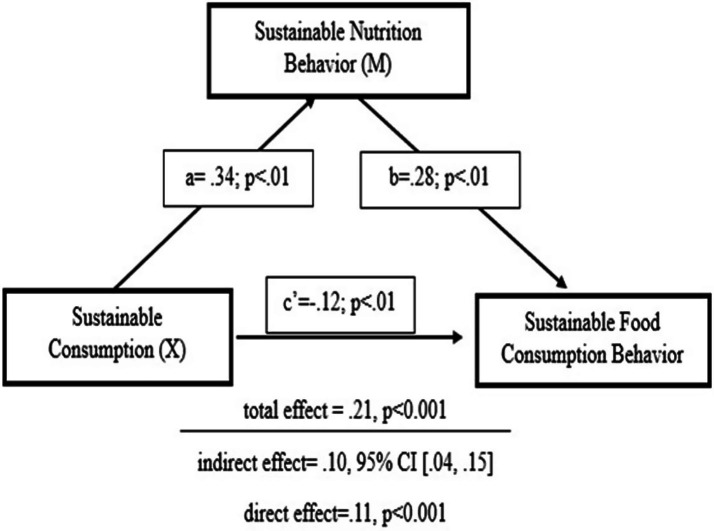
The mediation effect model of sustainable nutrition behavior.

**Figure 7:**
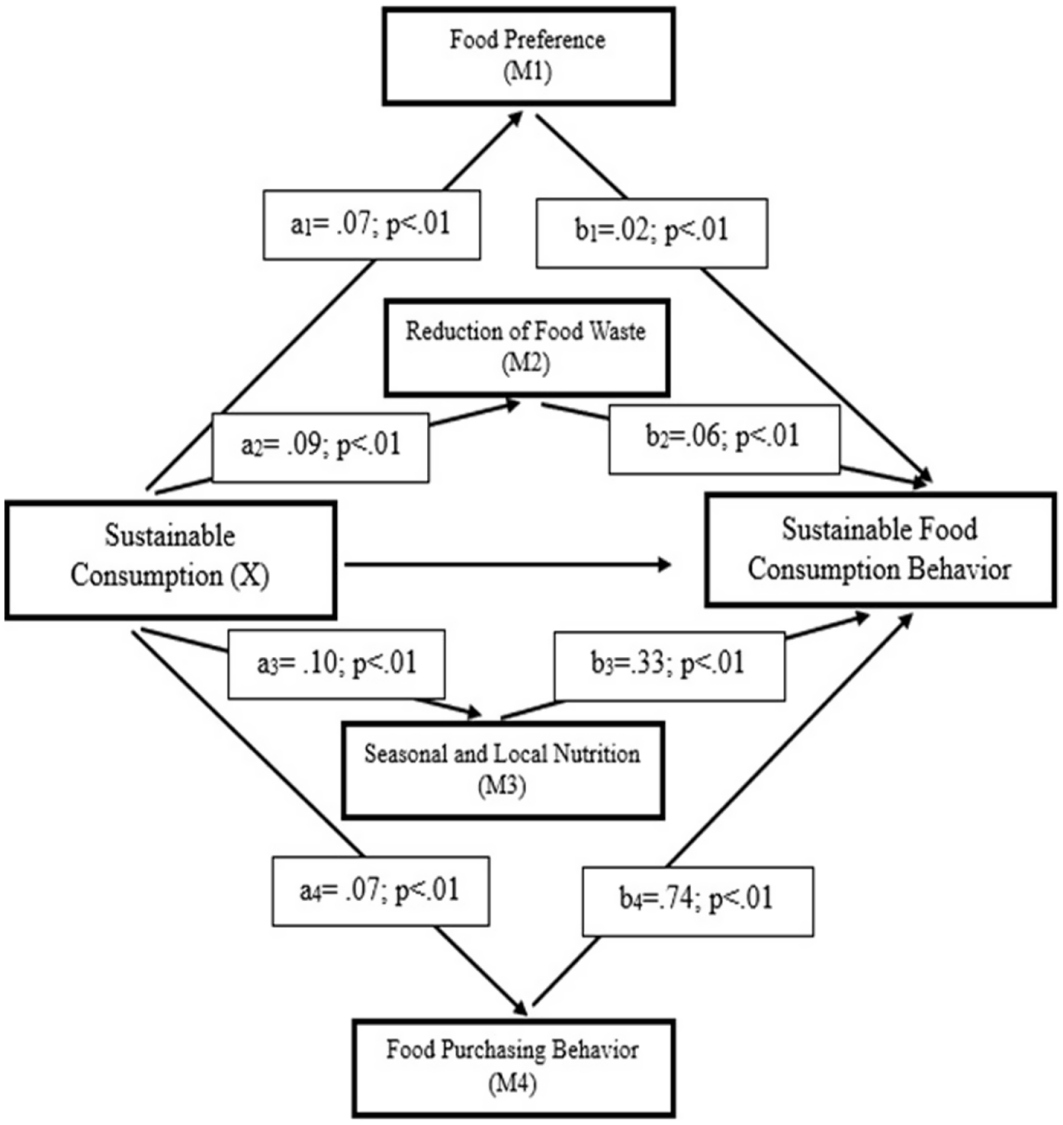
The mediation effect model of sustainable nutrition behavior’s sub-dimensions.

When the Figure 6, the mediation effect of the Sustainable Nutrition Behavior is examined, the findings show that the indirect effect of the Sustainable Consumption on Sustainable Food Consumption Behavior is significant, thus the Sustainable Nutrition Behavior mediates the relationship between the two variables (*b* = 0.10, % 95 BCA CI [0.04, 0.15]). As a matter of fact, as a result of the bootstrap analysis, it was determined that the corrected bias and accelerated confidence interval values (BCA CI) do not cover the value of zero (0) (55). The model related to the sub-dimensions of Sustainable Food Consumption Behavior is presented in Figure 7.

Table 10 shows that the model was found to be significant overall, *F*(5, 408) = 7.62, *p* < 0.001, and the variables explained 8.5% of the total variance in the dependent variable (*R*^2^ = 0.085). The independent variable (Sustainable Consumption) positively and significantly predicted the dependent variable (*b* = 0.198, *p* < 0.01). When the covariates were examined, only the age variable had a significant effect (*b* = 2.86, *p* < 0.001). However, the variables of gender, grade, and department did not show a significant effect on the dependent variable.

**Table 10:** Regression results for the total effect model predicting sustainable food consumption behavior.

Variable	*B*	SE	*t*	*p*	LLCI	ULCI
Constant	25.102	4.715	5.324	0.000	15.834	34.370
Sustainable consumption	0.198	0.056	3.538	0.000	0.088	0.308
Gender	0.723	1.073	0.673	0.501	−1.388	2.833
Age	2.860	0.658	4.347	0.000	1.567	4.154
Grade	−0.854	0.658	−1.297	0.195	−2.148	0.440
Department	0.141	0.531	0.266	0.790	−0.902	1.184

*R* = 0.292, *R*^2^ = 0.085, *F*(5, 408) = 7.62, *p* < 0.001.

## 4 Discussion

In this study conducted with students of Erciyes University Faculty of Sports Sciences, the effects of sustainable consumption on sustainable food consumption behavior were examined, especially within the framework of the mediating role of sustainable nutritional behavior, and the findings are stated in this section. Higher levels of sustainable consumption were observed to be associated with individuals’ sustainable food consumption behaviors. This finding aligns with the scale developed by Quoquab and Mohammad (39), which conceptualizes sustainable consumption through cognitive, affective, and behavioral dimensions. Awareness and positive attitudes are reflected at the behavioral level.

A weak but positive relationship was found between sustainable consumption and sustainable food consumption (*r* = 0.18, *p* < 0.001). Similarly, a weak yet significant correlation was observed between sustainable consumption and sustainable nutrition behavior (*r* = 0.16, *p* < 0.001). These findings indicate that individuals with higher awareness of sustainable consumption tend to adopt environmentally friendly dietary habits and general consumption behaviors.

A moderate and positive relationship was observed between sustainable nutrition behavior and sustainable food consumption (*r* = 0.51, *p* < 0.001). This suggests that sustainable dietary habits are associated with sustainable food choices (38, 40). Analyses of the sub-dimensions of sustainable nutrition behavior showed that each dimension was associated with sustainable food consumption. In particular, a tendency to prefer less processed, environmentally friendly, and plant-based foods was positively related to sustainable food consumption (56, 57).

Behaviors aimed at reducing food waste were moderately positively associated with sustainable food consumption (*r* = 0.42, *p* < 0.001). Similarly, the “Seasonal and Local Nutrition” sub-dimension was positively and significantly related to sustainable food consumption (*r* = 0.48, *p* < 0.001). These results indicate that preferences for locally and seasonally produced foods are associated with sustainable consumption (58–60). The “Food Purchasing Behavior” sub-dimension was significantly associated with sustainable food consumption (*r* = 0.52, *p* < 0.001), indicating a relationship between conscious purchasing decisions and sustainable consumption (61–64).

Analyses of sub-dimensions also revealed associations between food waste reduction and food preference behaviors with sustainable consumption (38, 42, 65, 66). Mediation analysis results supported the relationships between sustainable consumption, sustainable nutrition behavior, and sustainable food consumption. A positive association was found between sustainable consumption and sustainable nutrition behavior, and sustainable nutrition behavior was positively associated with sustainable food consumption (38, 61). Sustainable nutrition behavior partially supported the relationship between sustainable consumption and sustainable food consumption. At the sub-dimension level, the “Seasonal and Local Nutrition” dimension showed a strong association with sustainable food consumption (*b* = 0.74, *p* < 0.01). Behaviors aimed at reducing food waste also showed significant associations (*b* = 0.06, *p* < 0.01) (79).

The results of the mediation analysis revealed that sustainable nutrition behavior partially mediates the relationship between sustainable consumption and sustainable food consumption behavior. The bootstrapped indirect effect was statistically significant (a × b = 0.10, 95% BCa CI [0.04, 0.15], 5,000 resamples), with the confidence interval not crossing zero. This indicates that the mediation is robust and reliable. The effect size of the indirect effect suggests a moderate level of mediation. In practical terms, this means that sustainable consumption tendencies influence sustainable food consumption behavior not only directly, but also indirectly through individuals’ sustainable nutrition behavior.

The findings of this study indicated that the independent variable had a significant and positive effect on the dependent variable. When covariates were examined, only the age variable was found to be significant, while gender, grade, and department variables did not have a significant effect on the dependent variable. The effect of age is consistent with studies suggesting that individuals’ experience and maturation processes are reflected in various psychosocial outcomes (76). Conversely, the lack of effect of gender and similar demographic variables suggests that the mediation process operates independently of some contextual factors. The literature also suggests that the contribution of demographic variables to the mediation mechanisms may be limited (67–69). In this context, the findings indicate that the mediation effect strengthens, particularly when age is taken into account, but that it operates independently of other demographic variables.

These findings are consistent with the Theory of Planned Behavior (21), which suggests that attitudes, subjective norms, and perceived behavioral control are expressed through sustainable dietary behaviors, and that awareness is translated into actual consumption practices. However, the Theory of Planned Behavior has been used only partially, and future research should measure the full set of TPBs. Among Sport Science students, the results highlight relationships between sustainable consumption and dietary habits. Overall, the study presents a novel mediation model that describes how sustainable dietary behavior is associated with both attitudes and behaviors, offering insights for university programs and policy development.

## 5 Conclusion

In conclusion, the results indicate that higher sustainable consumption trends among Erciyes University Sports Sciences students are associated with improved sustainable dietary behaviors and sustainable food consumption. In this study, sustainable dietary behavior was determined to have a moderate partial mediating role. Sustainable dietary behavior is identified as a significant mediator, showing that students’ dietary habits are linked with sustainability-oriented attitudes and consumption patterns. These findings provide insights for sustainability awareness, plant-based and locally sourced meals, and waste reduction practices in Sports Sciences programs, highlighting their relevance for personal and professional sustainability behaviors.

## 6 Limitations of the study

This study has some limitations that should be considered. The sample was limited to students at Erciyes University’s Faculty of Sports Sciences, but Monte Carlo Simulation demonstrates the adequacy of the sample’s representativeness and generalizability. In the study, the sample group was selected using only the convenience sampling method. This study was conducted using a purely quantitative research design, without in depth qualitative interviews or direct observations of food consumption habits. Future research that includes more diverse samples in terms of age, occupation, and socioeconomic status could provide broader insights across different consumer profiles. Second, data were collected at a single point in time, which precludes definitive conclusions about causal relationships; longitudinal studies are needed to examine how behavioral changes evolve over time. Third, the study was conducted in the Turkish context, and the results may not be directly applicable to other countries or societies due to cultural, economic, or structural differences. Furthermore, the concept of sustainability is broad and multidimensional, but this study focused only on selected sub-dimensions such as dietary behavior, food waste, and purchasing behavior, excluding other components of sustainability. Furthermore, the study did not include carbon or water footprint calculations, and the results were based solely on participants’ self-reports, which may introduce subjective bias. In this study, only the Theory of Planned Behavior was used partially, and the full set of TPB should be measured in future research. The study was cross-sectional and entirely self-reported. Mediation here is purely statistical, accounting for potential biases such as common method variance and social desirability.

The self-report scales employed in this study involve several methodological limitations. First, due to social desirability bias, participants may refrain from disclosing their genuine thoughts, feelings, and behaviors, instead providing responses they perceive as more socially acceptable (70). This tendency is particularly problematic when addressing sensitive topics, as it can reduce the accuracy of the data (71). Moreover, because self-report techniques rely on individuals’ subjective perceptions, cognitive constraints (e.g., recall errors, selective memory, exaggeration) and motivational factors (e.g., the tendency to present oneself in an overly positive or negative manner) can introduce systematic biases (72). In addition, participants’ personality traits, current mood, attentional focus, and the way questions are formulated may all influence the consistency of responses (73, 74). Collectively, these factors can restrict the objectivity and internal validity of the findings, thereby limiting the generalizability of the results (75).

## 7 Practical recommendations

1. Nutrition education programs focusing on sustainability should be promoted and implemented specifically for students of the Faculty of Sports Sciences.
2. On-campus initiatives, such as local producer markets, anti-waste campaigns, and sustainable purchasing guides, should be established to engage students.
3. Educational policies should include interventions such as posters, announcements, and the development of cafeteria menus that exemplify sustainable consumption and nutrition, aiming to increase knowledge and encourage behavior change among students.
4. Experimental research designs should be employed to assess the effectiveness of educational programs, awareness campaigns, and other initiatives on students’ sustainable nutrition behaviors.

## 8 Recommendations for future research

1. This study was limited to university students. Future research could be applied to different age groups and compare their findings with the findings of our study (e.g., working adults, older adults).
2. Cross-sectional data were used in this study. Longitudinal studies can be conducted to track the evolution of behavioral changes over time, providing deeper insights into the persistence and evolution of sustainable eating behaviors. Group-time interactive studies can be carried out to monitor the change in practices that encourage sustainable consumption and sustainable eating behavior over time.
3. Experimental research designs to assess the impact of educational programs, campaigns, and awareness initiatives will be valuable for testing causal relationships.
4. What is holding back can be examined using qualitative methods such as focus group discussions or in-depth interviews to explore participants’ perceptions, attitudes, and perceived barriers to sustainable consumption and eating behaviors.
5. Future studies are recommended to adopt mixed methods approaches, integrating both quantitative and qualitative data, to better capture participants’ food choices, menu preferences, and environmental considerations.

## Data Availability

The raw data supporting the conclusions of this article will be made available by the authors, without undue reservation.

## References

[1] Barrett B. Health and sustainability co-benefits of eating behaviors: towards a science of dietary eco-wellness. Prev Med Rep. (2022) 28:101878. doi: 10.1016/j.pmedr.2022.101878, PMID: 35832638 PMC9272027

[2] Dönmez RÖ. Environmental awareness and sustainable consumption behaviors among nursing students. PeerJ. (2024). doi: 10.7717/peerj.17366 PMC11141558 38827292

[3] Barrera-Verdugo G. Influence of moral reasoning and environmental concern on sustainable food consumption behaviors. Sustain Dev. (2024). doi: 10.1016/j.clwas.2024.100164

[4] Lee Y Kim T Jung H. Effects of university students' perceived food literacy on ecological eating behavior towards sustainability. Sustainability. (2022) 14:5242. doi: 10.3390/su14095242

[5] Ekici EM Eroğlu FE Mengi Çelik Ö. The relationship between exercise health beliefs, sustainable healthy eating behaviors, and adherence to the mediterranean diet in female university students: EM Ekİcİ et al. Discover Public Health. (2025) 22:327. doi: 10.1186/s12982-025-00727-y

[6] Arrazat L Nicklaus S de Lauzon-Guillain B Marty L. Behavioural determinants of healthy and environmentally friendly diets in French university students. Appetite. (2024) 200:107532. doi: 10.1016/j.appet.2024.107532, PMID: 38815688

[7] Islam Q Khan SMF. Assessing consumer behavior in sustainable product markets: a structural equation modeling approach with partial least squares analysis. Sustainability. (2024) 16:3400. doi: 10.3390/su16083400

[8] Eraslan M Kır S Turan MB Iqbal M. Sustainability consciousness and environmental behaviors: examining demographic differences among sports science students. Sustainability. (2024) 16:10917. doi: 10.3390/su162410917

[9] Royet T Vors O Cece V Lentillon-Kaestner V. Education for sustainability and physical education: a systematic scoping review. Sport Educ Soc. (2024) 29:1000–18. doi: 10.1080/13573322.2024.2440886

[10] Xu X Sharma P Shu S Lin TS Ciais P Tubiello FN. Global greenhouse gas emissions from animal-based foods are twice those of plant-based foods. Nat Food. (2021) 2:724–32. doi: 10.1038/s43016-021-00358-x, PMID: 37117472

[11] Li Y Rao ND Wang X Chen C Qiu H. Reducing climate change impacts from the global food system. Nat Clim Chang. (2024) 14:1000–10. doi: 10.1038/s41558-024-02084-1

[12] Xipsiti M Rutherfurd-Markwick K Moughan PJ. Protein quality evaluation: FAO perspective. Front Nutr. (2024) 11:1446879. doi: 10.3389/fnut.2024.1446879, PMID: 39698253 PMC11654912

[13] Al-Nuaimi SR. Sustainable consumption and education for sustainability. Sustainability. (2022) 14:7255. https://www.mdpi.com/2071-1050/14/12/7255

[14] Scarborough P Allcott H McFadden D. Vegans, vegetarians, fish-eaters and meat-eaters in the UK show discrepant environmental impacts. Nat Food. (2023) 4:565–74. doi: 10.1038/s43016-023-00795-w 37474804 PMC10365988

[15] Gibas-Dorna M. Sustainable nutrition and human health as part of sustainable development. Nutrients. (2024) 16. doi: 10.3390/nu16020225, PMID: 38257118 PMC10819296

[16] Mahlaole ST van Niekerk E Mafongoya P. Education for sustainable development’s role in influencing sustainable consumption behaviour of youth in higher education. Discov Sustain. (2025) 6:83. doi: 10.1007/s44217-025-00498-3

[17] Fatemi SF. Influencing factors of adherence to sustainable diets: a systematic review based on behavioral theories. Front Sustain Food Syst. (2025). doi: 10.3389/fsufs.2025.1465622

[18] Çelik ÖM Akçakaya SD Ekici EM. Relationship between sustainable food literacy, organic food consumption and climate change awareness and worry in Türkiye. BMC Public Health. (2025) 25:2491. doi: 10.1186/s12889-025-22482-0, PMID: 40681983 PMC12273353

[19] Wu S-W Chiang P-Y. Exploring the mediating effects of the theory of planned behavior on the relationships between environmental awareness, green advocacy, and green self-efficacy on the green word-of-mouth intention. Sustainability. (2023) 15:12127. doi: 10.3390/su151612127

[20] Chapagain AK Hoekstra AY. The blue, green and grey water footprint of rice from production and consumption perspectives. Ecol Econ. (2011) 70:749–58. doi: 10.1016/j.ecolecon.2010.11.012

[21] Ajzen I. The theory of planned behavior. Organ Behav Hum Decis Process. (1991) 50:179–211. doi: 10.1016/0749-5978(91)90020-T

[22] Fishbein M Ajzen I. Predicting and changing behavior: The reasoned action approach. New York: Psychology Press (2010).

[23] Vermeir I Verbeke W. Sustainable food consumption among young adults in Belgium: theory of planned behavior and the role of confidence and values. Ecol Econ. (2008) 64:542–53. doi: 10.1016/j.ecolecon.2007.03.007

[24] Fischer D Böhme T Geiger SM. Measuring young consumers’ sustainable consumption behavior: development and validation of the YCSCB scale. Young Consum. (2017) 18:312–26. doi: 10.1108/YC-03-2017-00671

[25] Milfont TL Markowitz E. Sustainable consumer behavior: a multilevel perspective. Curr Opin Psychol. (2016) 10:112–7. doi: 10.1016/j.copsyc.2015.12.016

[26] Sharma R Jha M. Values influencing sustainable consumption behaviour: exploring the contextual relationship. J Bus Res. (2017) 76:77–88. doi: 10.1016/j.jbusres.2017.03.010

[27] Liang J Li J Cao X Zhang Z. Generational differences in sustainable consumption behavior among Chinese residents: implications based on sustainable consumption and lifestyle perceptions. Sustainability. (2024) 16:3976. doi: 10.3390/su16103976

[28] Wang P Liu Q Qi Y. Factors influencing sustainable consumption behaviors: a survey of the rural residents in China. J Clean Prod. (2014) 63:152–65. doi: 10.1016/j.jclepro.2013.05.007

[29] Nguyen TN Lobo A Greenland S. Pro-environmental purchase behaviour: the role of consumers' biospheric values. J Retail Consum Serv. (2016) 33:98–108. doi: 10.1016/j.jretconser.2016.08.010

[30] Giampietri E Verneau F Del Giudice T Carfora V Finco A. A theory of planned behaviour perspective for investigating the role of trust in consumer purchasing decisions related to short food supply chains. Food Qual Prefer. (2018) 64:160–6. doi: 10.1016/j.foodqual.2017.09.012

[31] Almoraie NM Alothmani NM Alomari WD Al-Amoudi AH. Addressing nutritional issues and eating behaviours among university students: a narrative review. Nutr Res Rev. (2024) 38:1–45. doi: 10.1017/S0954422424000088 38356364

[32] Kanosvamhira TP. Nourishing minds: understanding student dining preferences and perceptions of healthy eating in campus cafés. J Hunger Environ Nutr. (2025) 20:1–18. doi: 10.1080/19320248.2025.2506486, PMID: 40922668

[33] Sakamaki R Amamoto R Mochida Y Shinfuku N Toyama K. A comparative study of food habits and body shape perception of university students in Japan and Korea. Nutr J. (2005) 4:1–6. http://www.nutritionj.com/content/4/1/31 16255785 10.1186/1475-2891-4-31 PMC1298329

[34] Jeong MM Jung Y Koo DD. College students' perceptions of sustainability: a regional survey. J Build Constr Plan Res. (2015) 3:209–20. doi: 10.4236/jbcpr.2015.34021

[35] Kirby CK Zwickle A. Sustainability behaviors, attitudes, and knowledge: comparing university students and the general public. J Environ Stud Sci. (2021) 11:639–47. doi: 10.1007/s13412-021-00717-x

[36] Lanzini P Thøgersen J. Behavioural spillover in the environmental domain: an intervention study. J Environ Psychol. (2014) 40:381–90. doi: 10.1016/j.jenvp.2014.09.006

[37] Vermeir I Verbeke W. Sustainable food consumption: exploring the consumer “attitude–behavioral intention” gap. J Agric Environ Ethics. (2006) 19:169–94. doi: 10.1007/s10806-005-5485-3

[38] Garipoğlu G Koc BM Ozlu T. Behaviors scale towards sustainable nutrition: development and validity-reliability analysis. Nutr Food Sci. (2023). doi: 10.1108/NFS-09-2022-0335

[39] Quoquab F Mohammad J. Cognitive, affective, and conative domains of sustainable consumption: scale development and validation using confirmatory composite analysis. Sustainability. (2020) 12:7784. doi: 10.3390/su12187784

[40] Nguyen TTT Hetherington JB O'Connor PJ Malek L. Sustainable food consumption: sustainabil-ity-conscious consumers do not reduce food waste, but nutrition-conscious consumers do. Resour Conserv Recycl. (2025) 219:108296. doi: 10.1016/j.resconrec.2025.108296

[41] O'Neill C McCarthy MB O'Reilly S Alfnes F. Food interests, preferences and behaviours: a profile of the sustainable food consumer. Br Food J. (2023) 125:352–74. doi: 10.1108/BFJ-09-2022-0762

[42] Geiger SM Fischer D Schrader U. Measuring what matters in sustainable consumption: an integrative framework for selecting relevant behaviors. Sustain Dev. (2017) 26:18–33. doi: 10.1002/sd.1688

[43] McDermott MS Oliver M Svenson A Simnadis T Beck EJ Coltman T. The theory of planned behaviour and discrete food choices: a systematic review and meta-analysis. Int J Behav Nutr Phys Act. (2015) 12:162. doi: 10.1186/s12966-015-0324-z, PMID: 26715190 PMC4696173

[44] Hojjati M Mirzaei A Hemmati N Shamsabadi BG. Predicting consumers' intention and behavior towards industrial dairy products: a study based on an extended theory of planned behavior. Appl Food Res. (2025) 5:101129. doi: 10.1016/j.afres.2025.101129, ResearchGate+2foodandnutritionresearch.net+2

[45] Tutar H Erdem AT. Örnekleriyle bilimsel araştırma yöntemleri ve SPSS uygulamaları Seçkin, Ankara (2020).

[46] Büyüköztürk Ş. Akgün Ö. E. Demirel F. Karadeniz Ş. Çakmak E. K. (2015). Bilimsel araştırma yöntemleri

[47] Cohen J. Statistical power analysis for the behavioral sciences. 2nd ed Hillsdale, New Jersey: Lawrence Erlbaum Associates (1988).

[48] Green SB. How many subjects does it take to do a regression analysis? Multivar Behav Res. (1991) 26:499–510. doi: 10.1207/s15327906mbr2603_7, PMID: 26776715

[49] Maxwell SE Kelley K Rausch JR. Sample size planning for statistical power and accuracy in parameter estimation. Annu Rev Psychol. (2008) 59:537–63. doi: 10.1146/annurev.psych.59.103006.093735, PMID: 17937603

[50] Muthén LK Muthén BO. How to use a Monte Carlo study to decide on sample size and determine power. Struct Equ Modeling. (2002) 9:599–620. doi: 10.1207/S15328007SEM0904_8

[51] Tabachnick BG Fidell LS. Using multivariate statistics. 6th ed. Boston, MA: Pearson (2013).

[52] Akgün AA Kortunay N. Sürdürülebilir tüketim ölçeğinin Türkçeye uyarlanması: Geçerlik ve güvenirlik çalışması. Business Econ Res J. (2024) 15:419–433. doi: 10.20409/berj.2024.453

[53] Özen B. (2022) Sürdürülebilir Besin Tüketim Davranışı Ölçeği’nin Türkçe geçerlik-güvenirliği ve bazı sürdürülebilir diyet modelleriyle ilişkisinin incelenmesi (Yüksek lisans tezi, Hacettepe Üniversitesi Sağlık Bilimleri Enstitüsü). Available online at: https://tez.yok.gov.tr/ (Accessed July 24, 2025).

[54] George D Mallery P. IBM SPSS statistics 23 step by step. 13th ed. New York: Routledge (2016).

[55] Hayes AF. Introduction to mediation, moderation, and conditional process analysis: a regression-based approach. New York, NY: Guilford Press (2013).

[56] Kenger EB Özlü Karahan T Meral Koç B. Planet-friendly plates: the effect of a plant-based diet on sustainable eating behavior in a sample of Turkish adults. J Sci Food Agric. (2025) 105:3416–22. doi: 10.1002/jsfa.14102, PMID: 39725867

[57] Lea E Worsley T. Australians’ organic food beliefs, demographics, and values. Br Food J. (2005) 107:855–69. doi: 10.1108/00070700510629797

[58] Ismael AS Balogh P. Exploring the nexus between sustainable consumption behavior and organic food purchase: a comprehensive review. Appl Stud Agribusiness Commerce. (2023) 17:1–15. doi: 10.19041/APSTRACT/2023/2/11

[59] López LA Tobarra MA Cadarso MÁ Gómez N Cazcarro I. Eating local and in-season fruits and vegetables: carbon-water-employment trade-offs and synergies. Ecol Econ. (2022) 192:107270. doi: 10.1016/j.ecolecon.2021.107270

[60] Vargas AM de Moura AP Deliza R Cunha LM. The role of local seasonal foods in enhancing sustainable food consumption: a systematic literature review. Foods. (2021) 10:2206. doi: 10.3390/foods10092206, PMID: 34574316 PMC8465681

[61] Gallo T Pacchera F Cagnetti C Silvestri C. Do sustainable consumers have sustainable behaviors? An empirical study to understand the purchase of food products. Sustainability. (2023) 15:4462. doi: 10.3390/su15054462

[62] Ifeanyichukwu CD Nwaizugbo CI. Exploring critical factors influencing sustainable food consumption: a conceptual review. Eur J Business Innov Res. (2020) 8:32–42. doi: 10.37745/ejbir/vol8.no1.pp32-42.2020, PMID: 19121889

[63] Pšurný M Baláková I Stávková J Langr A. Perceived determinants of food purchasing behavior applicable for behavioral change toward sustainable consumption. Front Sustain Food Syst. (2024) 7:1258085. doi: 10.3389/fsufs.2023.1258085

[64] Shen X Xu Q Liu Q. Predicting sustainable food consumption across borders based on the theory of planned behavior: a meta-analytic structural equation model. PLoS One. (2022) 17:e0275312. doi: 10.1371/journal.pone.0275312, PMID: 36383540 PMC9668161

[65] Blanco-Penedo I García-Gudiño J Angón E Perea JM Escribano AJ Font-i-Furnols M. Exploring sustainable food choices, factors, and purchasing behavior in Spain's sustainable development goals era. Sustainability. (2021) 13:7397. doi: 10.3390/su13137397

[66] Gaiani S Rolle R Bucatariu C. Consumer-level food waste prevention and reduction towards sustainable diets In: Sustainable, editor. Diets: Linking nutrition and food systems. Wallingford, UK: CABI (2019). 89–98.

[67] Baron RM Kenny DA. The moderator–mediator variable distinction in social psychological research: conceptual, strategic, and statistical considerations. J Pers Soc Psychol. (1986) 51:1173–82. doi: 10.1037/0022-3514.51.6.1173, PMID: 3806354

[68] Hayes AF. Introduction to mediation, moderation, and conditional process analysis: A regression-based approach. 2nd ed New York, NY: Guilford Press (2018).

[69] Preacher KJ Hayes AF. Asymptotic and resampling strategies for assessing and comparing indirect effects in multiple mediator models. Behav Res Methods. (2008) 40:879–91. doi: 10.3758/BRM.40.3.879, PMID: 18697684

[70] Fisher RJ. Social desirability bias and the validity of indirect questioning. J Consum Res. (1993) 20:303–15. doi: 10.1086/209351

[71] Krumpal I. Determinants of social desirability bias in sensitive surveys: a literature review. Qual Quant. (2013) 47:2025–47. doi: 10.1007/s11135-011-9640-9

[72] Chan D. So why ask me? Are self-report data really that bad? In: Lance CE Vandenberg RJ, editors. Statistical and methodological myths and urban legends: Doctrine, verity and fable in the organizational and social sciences. New York, NY: Routledge (2009). 309–36.

[73] Paulhus DL Vazire S. The self-report method In: Robins RW Fraley RC Krueger RF, editors. Handbook of research methods in personality psychology. New York, NY: Guilford Press (2007). 224.

[74] Podsakoff PM MacKenzie SB Lee JY Podsakoff NP. Common method biases in behavioral research: a critical review of the literature and recommended remedies. J Appl Psychol. (2003) 88:879–903. doi: 10.1037/0021-9010.88.5.879, PMID: 14516251

[75] Tourangeau R Yan T. Sensitive questions in surveys. Psychol Bull. (2007) 133:859–83. doi: 10.1037/0033-2909.133.5.859, PMID: 17723033

[76] Mackinnon DP Fairchild AJ Fritz MS. Mediation analysis. Annu Rev Psychol. (2007) 58:593–614. doi: 10.1146/annurev.psych.58.110405.085542, PMID: 16968208 PMC2819368

[77] Sanchez LA Roa-Diaz ZM Gamba M Grisotto G Londono AM Mantilla-Uribe BP. What influences the sustainable food consumption behaviours of university students? A systematic review. Int. J. Public Health. (2021) 27:1–14. doi: 10.3389/ijph.2021.1604149 PMC8454891 34557062

[78] He J Sui D. Investigating college students’ green food consumption intentions in China: integrating the Theory of Planned Behavior and Norm Activation Theory. Front. Sustain. Food Syst. (2024) 8:1404465. doi: 10.3389/fsufs.2024.1404465

[79] Carvalho GP Costa-Camilo E Duarte I. Advancing health and sustainability: A holistic approach to food production and dietary habits. Foods. (2024) 13:3829. doi: 10.3390/foods13233829 39682901 PMC11640237

