# Supplementary material for: Sustainable dietary behavior and food choices among sport sciences students: a cross-sectional study within the theory of planned behavior
Source: Front Nutr. 2025 Oct 8;12:1689613. doi: 10.3389/fnut.2025.1689613 (PMC12540135; doi:10.3389/fnut.2025.1689613)
Supplement: Supplementary file 1 [file Table_1.pdf]

Appendix A

Exploratory factor analyses of the scales are given below.

Table 1

Sustainable Consumption Scale

Items	Cognitive Sustainable Consumption	Behavioral Sustainable Consumption	Affective Sustainable Consumption
Item 1	.829		
Item 2	.832		
Item 3	.815		
Item 4	.833		
Item 5	.866		
Item 6	.746		
Item 7	.751		
Item 8		.453	
Item 9		.495	
Item 10		.424	
Item 11		.551	
Item 12		.450	
Item 13		.681	
Item 14			.708
Item 15			.940
Item 16			.927

Table 2

Sustainable Nutrition Behavior Scale

Items	Food Preference	Reduction of Food Waste	Seasonal and Local Nutrition	Food Purchasing Behavior
Item 1	.491			
Item 2	.504			

Item 3	.595			
Item 4	.756			
Item 5	.518			
Item 6	.769			
Item 7		.533		
Item 8		.572		
Item 9		.696		
Item 10		.691		
Item 11		.730		
Item 12		.439		
Item 13		.635		
Item 14		.471		
Item 15		.621		
Item 16			.467	
Item 17			.419	
Item 18			.405	
Item 19			.498	
Item 20			.510	
Item 21			.606	
Item 22			.663	
Item 23			420	
Item 24				.505
Item 25				.530
Item 26				.540
Item 27				.425
Item 28				.737
Item 29				.844

Table 3

Sustainable Food Consumption Behavior Scale

Items	Purchasing Preferences	Dietary Preferences
Item1	.508	
Item 2	.908	
Item 3	.728	
Item 4	.847	
Item 5	.593	
Item 6		.485
Item 7		.432
Item 8		.662
Item 9		.727
Item 10		.733
Item 11		.643

Appendix B

Intra-scale correlation analyses of the sub-dimensions of the scales are given below.

Table 1

Sustainable Consumption Scale

	Cognitive Sustainable Consumption	Behavioral Sustainable Consumption	Affective Sustainable Consumption	Sustainable Consumption Scale Total
Cognitive Sustainable Consumption	1			
Behavioral Sustainable Consumption	.770**	1		
Affective Sustainable Consumption	.543**	.734**	1	

Sustainable Consumption Scale Total	.544**	.629**	.570**	1

**p<.001

Table 2

Sustainable Nutrition Behavior Scale

	Food Preference	Reduction of Food Waste	Seasonal and Local Nutrition	Food Purchasing Behavior	Sustainable Nutrition Behavior Scale Total
Food Preference	1				
Reduction of Food Waste	.645**	1			
Seasonal and Local Nutrition	.710**	.707**	1		
Food Purchasing Behavior	.668**	.703**	.757**	1	
Sustainable Nutrition Behavior Scale Total	.845**	.884**	.905**	.882**	1

**p<.001

Table 3

Sustainable Food Consumption Behavior Scale

	Purchasing Preferences	Dietary Preferences	Sustainable Food Consumption Behavior Scale Total
Purchasing Preferences	1		
Dietary Preferences	.735**	1	
Sustainable Food Consumption Behavior Scale Total	.965**	.887**	1

**p<.001
